# Graphene Oxide Hybridized nHAC/PLGA Scaffolds Facilitate the Proliferation of MC3T3-E1 Cells

**DOI:** 10.1186/s11671-018-2432-6

**Published:** 2018-01-11

**Authors:** Chunyong Liang, Yongchao Luo, Guodong Yang, Dan Xia, Lei Liu, Xiaomin Zhang, Hongshui Wang

**Affiliations:** 1 Research Institute for Energy Equipment Materials, Tianjin Key Laboratory of Materials Laminating Fabrication and Interface Control Technology, College of Materials Science and Engineering, Hebei University of Technology, Tianjin, 300130 China; 2 Institute for Advanced Materials, Jiangsu University, Zhenjiang, People’s Republic of China

**Keywords:** Graphene oxide, Poly(lactic-co-glycolic acid), Collagen, Nano-hydroxyapatite, Biodegradable porous scaffold, Bone tissue engineering

## Abstract

Biodegradable porous biomaterial scaffolds play a critical role in bone regeneration. In this study, the porous nano-hydroxyapatite/collagen/poly(lactic-co-glycolic acid)/graphene oxide (nHAC/PLGA/GO) composite scaffolds containing different amount of GO were fabricated by freeze-drying method. The results show that the synthesized scaffolds possess a three-dimensional porous structure. GO slightly improves the hydrophilicity of the scaffolds and reinforces their mechanical strength. Young’s modulus of the 1.5 wt% GO incorporated scaffold is greatly increased compared to the control sample. The in vitro experiments show that the nHAC/PLGA/GO (1.5 wt%) scaffolds significantly cell adhesion and proliferation of osteoblast cells (MC3T3-E1). This present study indicates that the nHAC/PLGA/GO scaffolds have excellent cytocompatibility and bone regeneration ability, thus it has high potential to be used as scaffolds in the field of bone tissue engineering.

**Electronic supplementary material:**

The online version of this article (10.1186/s11671-018-2432-6) contains supplementary material, which is available to authorized users.

## Background

Bone tissue engineering combining three-dimensional porous scaffolds and bone cells has been widely studied as an attractive approach in the treatment of malfunctioning or lost tissue [1]. Biodegradable scaffolds, which mimic the nature of the bone, play an important role for accommodating cells, controlling cell adhesion and proliferation, and facilitating bone regeneration [2]. Till now, various methods including electrospinning, integration of computational topology design (CTD) and solid free-form fabrication (SFF), and freeze-drying have been applied to fabricate different three-dimensional (3D) porous structures [3–7]. Electrospinning is able to make nanofibrous or microfibrous scaffolds with complex structures (aligned, spring-like fiber) and compositions [7]. However, the production efficiency of it is a bit low. The integration of CTD and SFF allows design of 3D anatomic scaffolds with porous architecture and better mechanical property. But this method requires strong professional knowledge [4]. Compared to these two methods, freeze-drying method allows fabricating porous structures with a much simpler process by the sublimation of frozen liquid phase under vacuum to fabricate a porous structure [8].

Nature bones possess complex hierarchical architecture with two main components, collagen and hydroxyapatite [9–11]. In bone tissue engineering, fabricating an ideal biomimicry of the bone extracellular matrix accommodating for cell adhesion and proliferation for the treatment of malfunction is still a challenge [12]. Nano-hydroxyapatite/collagen (nHAC)-based biodegradable scaffolds which are mimic of the natural bone could provide better biocompatibility, cell affinity, and bioresorbability [13]. However, the drawbacks of collagen, including the poor mechanical and rapid degrading properties, remain an obstacle for its application in bone tissue engineering [14]. Biodegradable aliphatic polymers, such as poly(lactic-co-glycolic) acid (PLGA), with high mechanical strength, outstanding biocompatibility, biodegradability, and good solubility in organic solvents, are ideal compensated material constructing 3D porous scaffolds for bone tissue engineering [15, 16]. A hybrid porous scaffold containing collagen and synthetic polymers combines the advantages of collagen and polymers and overcome their weaknesses, which is extensively used for bone repair and regeneration [17–19]. For instance, Liao et al. have developed a bone scaffold prepared by nHAC and poly(lactic acid) (PLA) to promote bone regeneration [17]. Niu et al. have fabricated nHAC/poly(L-lactic acid)/chitosan microspheres composite scaffolds for enhancing osteoblast proliferation [19].

Recently, graphene oxide (GO), a novel carbon sheet with one-atom thickness [20, 21], have attracted great interest in biological field because it owns good biocompatibility. The GO hybridized scaffolds are able to enrich both the mechanical property of the scaffold and the cellular behaviors, such as cell spreading and proliferation [22, 23]. Luo et al. reported that the incorporation of GO into PLGA nanofibrous enhanced proliferation and osteogenic differentiation of mesenchymal stem cells (MSCs) [20]. Jing et al. reported that the addition of 1.0 wt% GO into the thermoplastic polyurethane could facilitate the Swiss mouse fibroblasts cell proliferation [24]. Compared with adding the chemical cross-linking agents (genipin, glutaraldehyde, carbodiimide, etc*.*) [25, 26], which have certain cytotoxicity, to improve the mechanical property of composite scaffolds, the small amount of GO hybridized scaffolds show good biocompatibility. Therefore, the hybridization of the GO and the nHAC/PLGA could be a novel artificial scaffold for bone tissues.

In this study, the porous nano-hydroxyapatite/collagen/poly(lactic-co-glycolic acid) /graphene oxide (nHAC/PLGA/GO) scaffolds, which contains different weight ratio of GO (0.0, 0.5, 1.0, and 1.5 wt%) have been fabricated and characterized. The hybridization scaffolds show porous structures. The addition of GO modifies the hydrophilic property and the mechanical property of the hybridization scaffolds. To investigate the effect of the nHAC/PLGA/GO scaffold on bone tissue engineering, the MC3T3-E1 cells were cultured on the porous hybridization scaffolds. The results show that the 1.5 wt% GO-doped hybridization scaffolds facilitate cell adhesion, growth, and proliferation, further indicating nHAC/PLGA/GO scaffold can be considered as a promising candidate in bone tissue engineering.

## Results and Discussion

### Structure of nHAC/PLGA/GO Composite Scaffolds

**Fig. 1:**
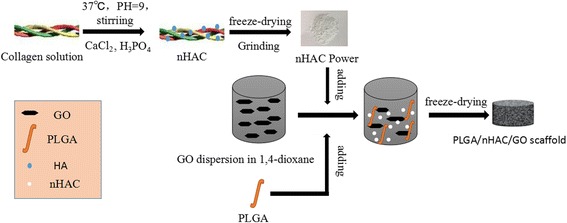
Schematic diagram of the fabrication process for nHAC/PLGA/GO scaffolds

**Fig. 2:**
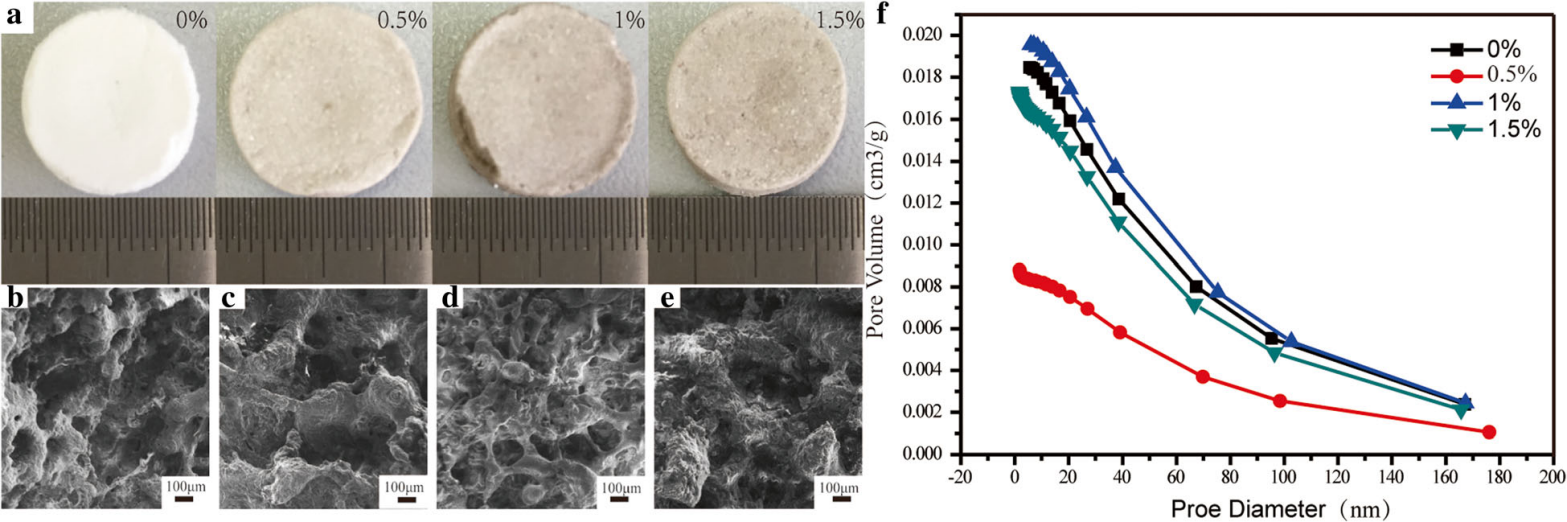
**a** Optical image of the synthesized nHAC/PLGA/GO scaffolds with different amount of GO. **b**–**e** SEM images of **b** nHAC/PLGA, **c** nHAC/PLGA/GO (0.5 wt%), **d** nHAC/PLGA/GO (1.0 wt%), and **e** nHAC/PLGA/GO (1.5 wt%) scaffolds. **f** Hole distribution of nHAC/PLGA/GO(0, 0.5, 1.0, and 1.5 wt%)

Figure 1 illustrates the fabrication process of nHAC/PLGA/GO scaffolds. The details of the fabrication process are shown in the experimental section. The nHAC was synthesized prior to fabricate the nHAC/PLGA/GO composite scaffolds. The scanning electron microscope (SEM) image of the nHAC powder shows its nanostructure. The corresponding energy-dispersive X-ray spectroscopy (EDS) spectra of nHAC is also shown (Additional file 1: Figure S1), which reveals the presence of Ca, Cu, P, C, and O. Copper signals should be contributions of the supporting samples. Thus, the nHAC is composed of Ca, P, C, O, and the Ca:P molar ratio of nHAC powder is 1.41, which is lower than that of hydroxyapatite (HA) (1.66). This indicates that the synthesized HA is calcium deficiency type [27], which will lead to reduction in the hardness, elastic modulus, and toughness in nHAC. To increase the mechanical properties of the composite scaffold, the PLGA and GO were added into the nHAC powder. The optical overview of the fabricated nHAC/PLGA/GO scaffolds with different amount of GO are shown in Fig. 2a. The sample is a cylinder with a diameter of 14 mm. It is apparent that the composite scaffolds without GO is in white color. As the increasing of the GO, the composite scaffolds become more and more dark. The detailed morphologies of different nHAC/PLGA/GO scaffolds are revealed by SEM (Fig. 2b–e). It significantly shows that all the scaffolds form porous structures and the surfaces of four different scaffolds are pretty rough. In order to characterize the information of these holes, we used an automatic surface area and porosity tester to evaluate. The results of the hole distribution was shown in Fig. 2f. The size of the four scaffold holes is between 0 and 200 nm. And the number of the holes which dozens of nanometers are more than those with a few hundred nanometers in four scaffolds. It has been reported that porosity of biomaterial scaffolds is non-trivial for bone formation in vitro and in vivo [28]. To optimize integration into surrounding tissue, scaffolds for osteogenesis should mimic bone morphology, structure, and function [4]. Thus, the 3D porous structure of nHAC/PLGA/GO composite scaffolds is critical for bone regeneration. The large-scale SEM images of the four composite scaffolds are also shown (Additional file 1: Figure S2), which illustrates the overview structures of different surfaces.

### Physicochemical and Mechanical Characterizations of nHAC/PLGA/GO Composite Scaffolds

**Fig. 3:**
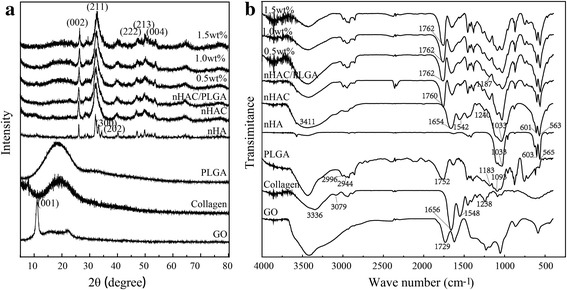
**a** XRD and **b** FT-IR spectra of different components

The mechanism of the synthesize process can be revealed by the X-ray diffraction (XRD) and Fourier transform infrared spectroscopy (FT-IR) spectra of various single substance and composites (Fig. 3). As indicated in Fig. 3a, the inorganic phase was determined as HA according to the powder diffraction file (PDF card no. 09–0432) since no peaks from other Ca-P materials were present in the XRD pattern. Compared with nHA, the broaden diffraction peaks of nHAC implied a small grain size and low crystallinity. Similar to nHAC, the pattern of nHAC/PLGA/GO with different amount of GO also had low crystallinity. However, the peak of GO did not appear in the nHAC/PLGA/GO composites, which might because of the few amount of GO compared to the bulk. Figure 3b shows the FT-IR spectra of various single substance and composites. From Fig. 3b, the typical bands for collagen can be observed, such as N–H stretching at 3336 cm^−1^ for amide A; C–H stretching at 3079 cm^−1^ for amide B; C=O stretching at 1656 cm^−1^ for the amide I; N–H deformation at 1548 cm^−1^ for the amide II and absorption peak at 1238 cm^−1^ for amide III. As the formation of nHAC, the amide A moves from 3336 cm^−1^ to around 3411 cm^−1^, the amide B was weakened, the amide I, amide II, amide III move from 1656, 1548, and 1238 cm^−1^ to 1654, 1542, and 1240 cm^−1^, respectively. Thus, it confirms the chemical reaction between collagen and HA. Additionally, the peaks at 1033, 601, and 563 cm^−1^ are the typical peaks for (PO4)^3−^ group, which indicates the newly formation of HA on the collagen because of the merchandized HA only possessing characteristic peaks of (PO4)^3−^ at 1033, 603, and 565 cm^−1^. The characterized peaks of PLGA around 2996 and 2944 cm^−1^ were assigned to −CH_2_, 1752 cm^−1^ was assigned to C=O, 1183 and 1093 cm^−1^ were assigned to C–O, are clearly seen. Compared with PLGA scaffold, the peaks of nHAC/PLGA scaffold move from 1752 and 1183 cm^−1^ to 1760 and 1187 cm^−1^, respectively, which demonstrate the chemical reaction between PLGA and nHAC power. Compared with nHAC/PLGA scaffold, the peaks of GO-doped nHAC/PLGA scaffolds moved from 1760 to 1762 cm^−1^; there is a red shift occurred which demonstrates the chemical reaction between GO and nHAC/PLGA.

**Fig. 4:**
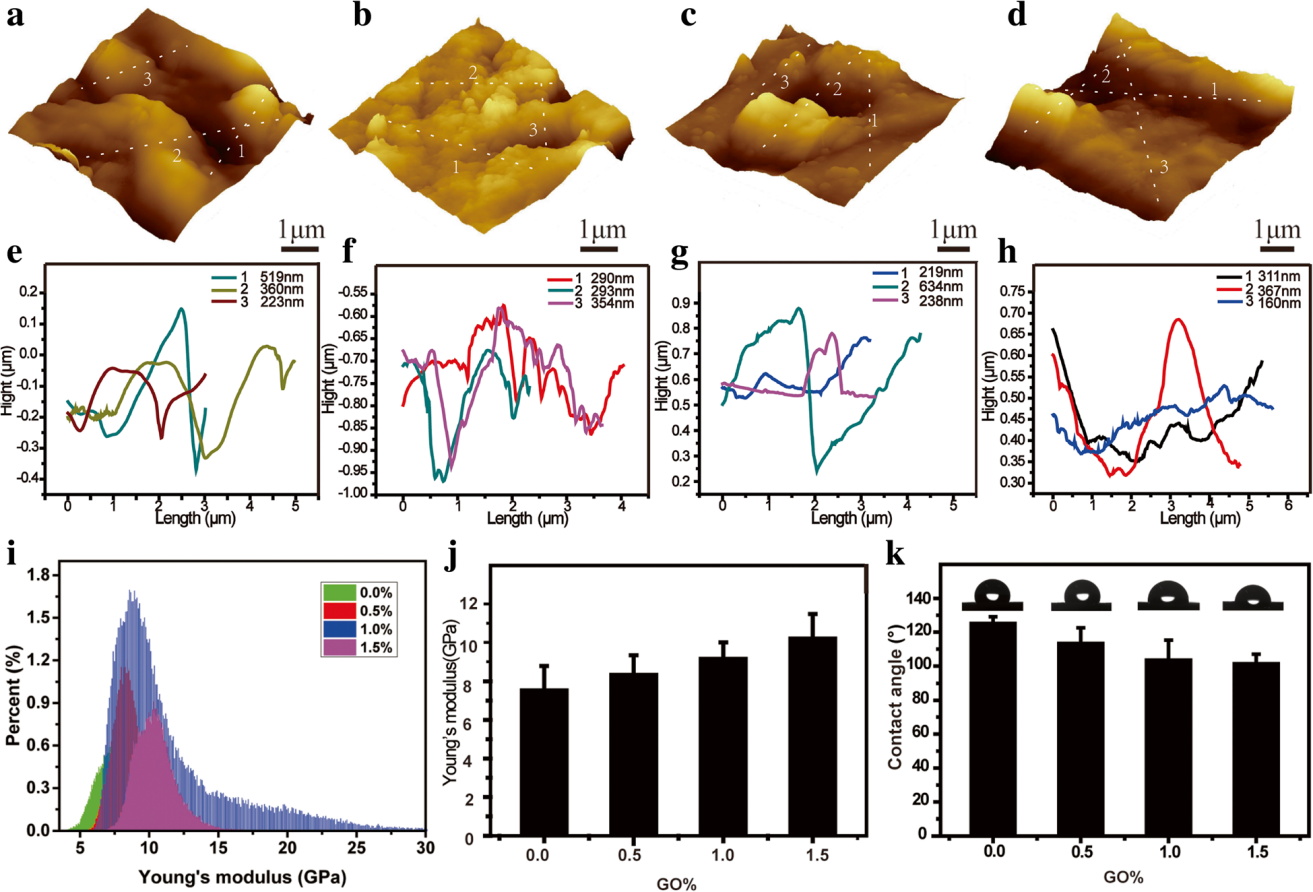
AFM images of **a** nHAC/PLGA, **b** nHAC/PLGA/GO (0.5 wt%), **c** nHAC/PLGA/GO (1.0 wt%), and **d** nHAC/PLGA/GO (1.5 wt%) scaffolds. **e**–**h** Line profiles derived from the morphology images. **i** Stiffness distribution of the four different scaffolds measured by QNM-AFM. **j** The bar chart of Young’s modulus versus the GO amount. **k** The corresponding contact angles of four kinds of scaffolds measured by the sessile drop method

The nanostructure and mechanical property of the nHAC/PLGA/GO scaffolds with different GO amount were characterized by quantitative nano-mechanical atomic force microscope (QNM-AFM) [29–34], which is able to provide the morphology and the stiffness spontaneously and is widely used to detect the mechanical properties of various materials, including bone [30], teeth [35], cornea [36], etc. Figure 4a–d shows the tomography of four kinds of composite scaffolds. Due to the limitation of the scanning areas, AFM images only shows local surface structure. Thus, the porous structure is not obvious. However, the AFM images also show rough surface morphology similar with the SEM images. Roughness has important effect on cell proliferation and differentiation. The surface with rough surface was beneficial to the cell proliferation and differentiation [37–39]. The line profiles (Fig. 4e–h) derived from the morphology (Fig. 4a–d) show the maximum height differences alone different line direction. It is clearly shown that the maximum height differences range from ~ 200 to ~ 600 nm. The corresponding stiffness distribution (Fig. 4i) shows the Young’s modulus of four different scaffolds are 7.53 ± 1.25, 8.34 ± 1.00, 9.15 ± 0.85, and 10.20 ± 1.28 GPa, respectively. To clearly show the stiffness differences, the corresponding bar chart is also given (Fig. 4j). Although the Young’s modulus of the nHAC/PLGA/GO scaffolds with a few GO amount difference are not significantly different, for example, the nHAC/PLGA/GO with the GO amount of 0.0 and 0.5 wt% (7.53 ± 1.25 and 8.34 ± 1.00 GPa), of 0.5 and 1.0 wt% (8.34 ± 1.00 and 9.15 ± 0.85 GPa), of 1.0 and 1.5 wt% (9.15 ± 0.85 and 10.20 ± 1.28 GPa), the Young’s modulus of the nHAC/PLGA/GO scaffolds with a bit large GO amount difference (0.0 wt% and 1.5 wt%) are significantly different (7.53 ± 1.25 and 10.20 ± 1.28 GPa). This indicates that the mechanical property of the nHAC/PLGA/GO scaffold increase with the increasing GO amount.

The hydrophilicity of the scaffolds plays a key role in interacting with cells. The addition of GO not only increases the mechanical property of the composite scaffolds, but also changes the hydrophobicity of four kinds of scaffolds. Figure 4f shows the contact angles of different nHAC/PLGA/GO scaffolds. The contact angles of the nHAC/PLGA scaffolds was ~ 125.1° while for nHAC/PLGA/GO with different GO amount (0.5. 1.0, and 1.5 wt%) are ~ 113.4°, ~ 103.4°, and ~ 101.4°, respectively. As the increasing of the GO amount, the contact angles of the composite scaffolds decrease slightly because of both the hydroxyl groups and the negatively charged groups, such as carboxylic acid groups on the GO surface [40]. Thus, GO can provide remarkable bioactivity to 3D nHAC/PLGA scaffolds.

In general, scaffolds for tissue engineering not only require exhibition of biocompatible morphology and properties, but also porous structure and physical strength [41]. The freeze-dried 3D nHAC/PLGA/GO scaffolds possess porous structure because of the sublimation of solvent. The functional groups, including hydroxyl (OH), epoxy (C-O-C), and carboxyl (COOH) species on scaffolds surfaces [40] induces good hydrophilicity. The addition of PLGA and GO provides sufficient physical strength. Thus, the 3D nHAC/PLGA/GO scaffolds could be a promising candidate for tissue engineering.

### Cell Culture

**Fig. 5:**
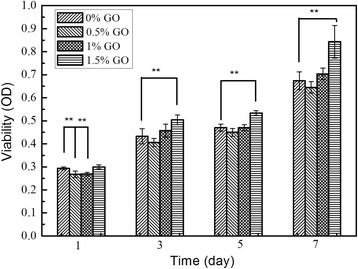
Comparison of the MC3T3-E1 cells on different scaffolds surfaces; double asterisks indicate *p* < 0.01, number of samples *N* = 4

It is well known that the scaffolds used for bone tissue should be biocompatible, cell proliferative, and exclusive from immune response [21]. The nHAC/PLGA/GO, which contains the components of natural bone (collagen and HA) and possesses suitable mechanical property and hydrophilicity, should be an ideal candidate for bone tissue engineering. To investigate the cell proliferation of these scaffolds, the MC3T3-E1 osteoblast cells were cultured in this work. Figure 5 shows the cell viability versus culture time evaluated by cell counting kit-8 (CCK-8) assay. The proliferation of cells was consistently increased during the whole culture period for all groups. More specifically, the cell proliferation of MC3T3-E1 on nHAC/PLGA/GO (0.5 and 1.0 wt%) scaffolds are significantly decreased at day 1, while that on nHAC/PLGA/GO (1.5 wt%) scaffolds is similar to that on nHAC/PLGA scaffolds. As time increases, the cell proliferation of MC3T3-E1 on nHAC/PLGA/GO (1.5 wt%) scaffolds is significantly increased at days 3, 5, and 7. However, the cell proliferation of MC3T3-E1 on nHAC/PLGA/GO (0.5 and 1.0 wt%) scaffolds is not significantly different compared to that on nHAC/PLGA scaffolds.

**Fig. 6:**
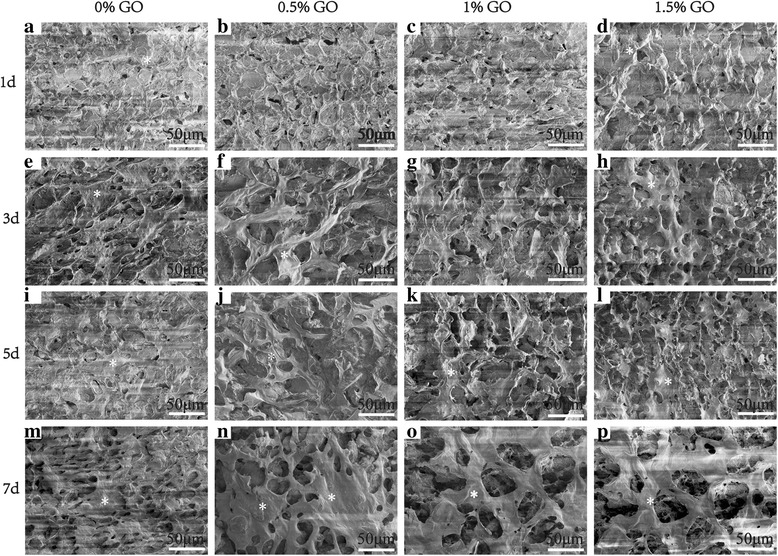
**a**–**p** SEM images of MC3T3-E1 cells cultured on four different scaffolds for 1, 3, 5, and 7 days. Scale bars are 50 μm in all images.The white asterisk represents the MC3T3-E1 osteoblast cells

The evidence of cell growth, proliferation on different scaffolds was also captured by SEM. Figure 6 shows the surface morphology of osteoblast cells on four different scaffolds after been cultured for 1, 3, 5, 7 days, respectively. At day 1, all the cells are evenly and isolated distributed on four different scaffolds. As time continues (days 3, 5, and 7), all groups of cells grow, proliferated, and started to integrate on different scaffolds, forming a large layer of cells. Compared with the cell morphologies on different scaffolds, the cells on nHAC/PLGA/GO scaffolds surfaces were much larger and stretched than that on the surface of nHAC/PLGA scaffolds. There is no significant difference in the situation of cells spreading, adhesion between the cells on nHAC/PLGA/GO with different amount (0.5, 1.0, and 1.5 wt%) according to the SEM images.

### Cytotoxicity Test

**Fig. 7:**
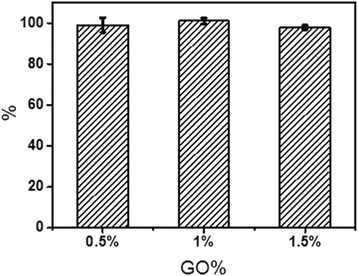
The relative activity of HIH-373 cells in nHAC/PLGA(0.5, 1, 1.5 wt%) relate to nHAC/PLGA

The cytotoxicity of GO is an essential concern, for its application in biology field. So we evaluate the cytotoxicity of the four scaffolds in the time of 24 h. The results were shown in Fig. 7. The cell vitality of fibroblasts cells (NIH-3T3) in nHAC/PLGA contain 0.5,1, and 1.5% GO is 99,101.11, and 97.86% relate to nHAC/PLGA, which have no significantly difference than nHAC/PLGA, indicating that the increase in graphene oxide is safe at 0–1.5%.

Table 1 summarized the mechanical properties and cell culture properties of four kinds of composite scaffolds. As the increasing of the GO, the Young’s modulus of the scaffolds increase accordingly. However, only the mechanical properties of nHAC/PLGA and nHAC/PLGA/GO (1.5 wt%) are distinctly different. The cell viability of four kinds of scaffolds show the same trend with the mechanical property, that is to say the OD values of all groups increase with the increasing cell culture time, but only the nHAC/PLGA and nHAC/PLGA/GO (1.5 wt%) groups show significant difference. This indicates that the mechanical property of the scaffolds is closely related to the cell culture property. The results may be because that tissue cells can feel and respond to the stiffness of their substrates [42–45]. Tuning the mechanical properties of the substrates can promote cellular responses affecting cell-surface interactions along with cell growth and viability [46–49]. Haugh et al. found that the stiffness of the scaffolds enhanced the activity of MC3T3-E1 cells (cell proliferation and migration) [50]. Engler et al. demonstrated that an important physical factor in the response of many cell types was substrate stiffness [51]. They found that smooth muscle cells derive from rat aorta (A7R5 line), like other anchorage dependent cells, spread more and organize their cytoskeleton and focal adhesions much more on ‘stiff’ substrates than on ‘soft’ substrates. The mechanical property not only affects the cell behaviors but also the tissue activities. Duncan et al. studied the mechanotransduction and the mechanical strain of functional response on bone. They found that mechanical loading can inhibit bone resorption and increase bone formation in vivo [52]. Therefore, the stiffest nHAC/PLGA/GO (1.5 wt%) scaffolds could promote the proliferation of MC3T3-E1 cells.

**Table 1:** Young’s modulus and OD values of different PLGA/nHAC/GO scaffolds

Young’s modulus and OD values	PLGA/nHAC	PLGA/nHAC/GO (0.5 wt%)	PLGA/nHAC/GO (1.0 wt%)	PLGA/nHAC/GO (1.5 wt%)
Young’s modulus	7.53 ± 1.25 (GPa)	8.34 ± 1.00 (GPa)	9.25 ± 0.85 (GPa)	10.20 ± 1.28 (GPa)
OD (1 day)	0.294 ± 0.006	0.268 ± 0.014	0.269 ± 0.007	0.300 ± 0.008
OD (3 days)	0.433 ± 0.033	0.406 ± 0.018	0.458 ± 0.028	0.505 ± 0.021
OD (5 days)	0.470 ± 0.015	0.450 ± 0.017	0.470 ± 0.013	0.534 ± 0.010
OD (7 days)	0.674 ± 0.039	0.645 ± 0.025	0.704 ± 0.026	0.843 ± 0.071

The cytotoxicity of GO is an essential concern for its application in biology field. Till now, two arguments have been arisen. One claim the GO would induce cytotoxicity and its effect is concentration dependent. For example, Chatterjee, et al. reported the toxic response with differential dose dependency for GO [53]. Pinto, et al. reported that only low concentrations of GO may be incorporated safety in PLA to facilitate cell adhesion and proliferation [6]. The others state that even higher amount of GO would have good biocompatibility and enhance both mechanical properties of the substrates and the cellular behaviors. Shin, et al. studied the C2C12 skeletal myoblasts were enhanced on PLGA-GO-collagen hybrid matrices than PLGA, PLGA-collagen matrices [54]. And Luo, et al. reported the GO-doped PLGA nanofiber scaffolds can enhance the osteogenic differentiation of MSCs [22]. In this study, the GO was selected based on the first argument. The limited amount is added into the composite scaffolds for non-cytotoxicity and enhanced mechanical property. The conjugation of GO into nHAC/PLGA scaffolds significantly enhanced cell growth, proliferation. Although the cell number on both nHAC/PLGA and nHAC/PLGA with small amount of GO, for example, nHAC/PLGA/GO (0.5 wt%), is more or less the same, the number of cells on nHAC/PLGA/GO (1.5 wt%) scaffold was higher than that on the nHAC/PLGA scaffolds. These results indicate the nHAC/PLGA/GO scaffolds are biofunctional with the ability of enhancing the growth and proliferation of MC3T3-E1 cells. Therefore, the excellent biocompatibility and biofunctionality allows nHAC/PLGA/GO to be employed as effective scaffolds for bone regeneration.

The nature of the biomaterial and the fabrication process are very important to scaffold properties [28]. So far, the biomaterials have been extensively studied, including metals [55], ceramics [56], glass [57], chemically synthesized polymers [58], natural polymers [59], and combinations of these materials to form composites [60]. Changing components of composite scaffolds will induce the scaffolds property. For instance, to fabricate the biomimic scaffold of natural bone, the type I collagen is been used in this study. Currently, the collagen family includes more than 20 different types of collagen existing in the skin, bone, cartilage, etc. Replacing type I collagen with other types, it is possible to fabricate different composite scaffolds for different purpose. For example, collagen type II is one of the fibril-forming collagens and the predominant type of collagen in cartilage. Coordinating collagen type II into the scaffolds may be able to facilitate cartilage bone regeneration [61]. In addition, the collagen with proper annealing may further strength the scaffolds, which may induce a new composite material with functional structures. Besides the nature of biomaterials, the processing also determines the functional of scaffolds, such as different processing methods. Material chemistry and processing determines the maximum functional properties as well as how cells interact with the scaffold. Scaffolds of properties and requirements in bone tissue engineering have been extensively investigated, including degradation [62], mechanical properties [63], cytokine delivery [64], and combinations of scaffolds and cells [65].

## Conclusions

In summary, nHAC/PLGA/GO scaffolds with different amount of GO (0.0, 0.5, 1.0, and 1.5 wt%) were fabricated by freeze-drying method. The fabricated nHAC/PLGA/GO scaffolds show porous structure. Furthermore, the mechanical property and the hydrophilicity of the scaffolds are enhanced because of the addition of PLGA and GO. The in vitro study shows the porous scaffolds facilitate the cell adsorption, growth, and proliferation. These nHAC/PLGA/GO scaffolds could be a promising candidate for bone tissue applications.

## Methods

### Materials

The purified lyophilized type 1 collagen was obtained from Tianjin Saining Biological Engineering Technology Co., Ltd. PLGA with lactide:glycolide ratio of 75:25 and Mw of 95,000 g/mol was purchased from Shandong Medical Appliance Factory (China). GO was purchased from Shanghai Aladdin biochemical Polytron Technologies Inc. MC3T3-E1 osteoblast cells were provided by cell bank of Shanghai Chinese Academy of Sciences. Fetal bovine serum (FBS), antibiotic-antimycotic, CCK-8, and Dulbecco’s modified Eagle media (DMEM) were accessed from Tianjin Nobuo Letter Technology Co., Ltd. 1,4-dioxane, phosphate buffered saline (PBS, 0.1 M, PH 7.4), and all other chemicals were analytical grade and used as received with no further purification.

### Preparation of the nHAC Power and nHAC/PLGA/GO Composite Scaffolds

The method of compositing nHAC powder has been reported previously [66–68]. Briefly, collagen was dissolved in acetic acid (0.5 mol/L) forming a solution with the concentration of 4 g/L. The CaCl_2_ and H_3_PO_4_ (Ca/*P* = 1.66) solutions were then added separately by drops. The dropping rate is 100 drops per minute. The solution was gently stirred and titrated at 37 °C with ammonia solution to pH 9. After 24 h, the nHAC deposition was harvested by centrifugation and freeze-drying. For the preparation of nHAC/PLGA/GO composite scaffolds, GO was evenly dispersed in dioxane by using an ultrasonic cell crusher, forming a final concentration of 0.0, 0.5, 1.0, and 1.5 g/L, respectively. The PLGA was then added into GO solutions, forming a final concentration of 10% (m/v). The GO/PLGA solutions were then stirred gently at room temperature for 12 h. The final solution was formed by adding the nHAC power into the GO/PLGA solution at a 1:1 nHAC:PLGA weight ratio. The formed nHAC/PLGA/GO solution was then stirred and ultrasonicated for 4 h. After frozen at − 20 °C overnight, the nHAC/PLGA/GO composite scaffolds were obtained by lyophilizing to remove dioxane.

### Characterizations

The composite scaffolds were coated with gold and were observed under a SEM (JSM-7100F). We spray gold for 20 s for preparation of electron microscopy samples. The topography and the mechanical properties of the matrices were characterized by atomic force microscopy (AFM, Multimode VIII, Bruker, Germany) in air. Image analysis was performed using Gwyddion and Nanoscope Analysis Software. Compositional analysis of the nHAC/PLGA/GO composite scaffolds was performed by a FT-IR spectrophotometer (VECTOR22, Bruker, Germany). All spectra were recorded in absorption mode in the wavelength range of 1000–2200 cm^−1^ with a resolution of 4.0 cm^−1^ and 16-times scanning. The contact angles of the samples were measured using a contact angle measurement system by the sessile drop method (EasyDrop, model DAS30, kruss, Germany). The XRD patterns were measured using the X-ray diffractometer (D8 DISCOVER). The Cu-Kα radiation (λ = 0.154 nm) is 40 kV and 30 mA. The scan rate of the measurements is 8°min^−1^ over the 2θ range of 5–80° at RT. The porosity of the scaffolds were measured by an automatic surface area and porosity analyzer (ASAP 2460, Micromeritics, GA, USA).

### Cell Culture

MC3T3-E1 osteoblast cells were incubated in DMEM supplemented with 10% FBS and 3% antibiotic-antimycotic solution at 37 °C and 5% CO_2_ in a cell incubator. The initial attachment and proliferation were tested by using CCK-8 according to the manufacturer’s instruction, in which the number of viable cells was directly proportional to the metabolic reaction products obtained in the CCK-8 assay [69]. Briefly, the MC3T3-E1 osteoblast cells were seeded at a density of 2.5 × 10^4^ cells per well on the nHAC/PLGA, nHAC/PLGA/GO (0.5 wt%), nHAC/PLGA/GO (1.0 wt%), and nHAC/PLGA/GO (1.5 wt%) matrices embedded in 48-well cell culture plate. The cells were incubated with the CCK-8 solution in the last 2 h of the culture periods (1, 3, 5, and 7 days) for the proliferation at 37 °C in the dark. The absorbance was measured at the wavelength of 450 nm using an ELISA reader (DNM-9602).

The cell samples for SEM measurement were fixed with formaldehyde, and the specimens were then dehydrated through a graded series of ethanol (30, 50, 75, 95, and 100%) for 15 min at each concentration. Then, the samples were drying by critical point drying was allowed to occur with a carbon dioxide analyzer (Hitachi, HCP-2). Finally, the samples with gold coating were observed by SEM.

### Cytotoxicity Test

The fibroblasts cells concentration was adjusted to 1 × 10^4^/ml and was inoculated into 96-well plates at 200 ul per well. Then, the well plates were incubated at 37 °C in a 5% CO_2_ incubator for 24 h. The samples (nHAC/PLGA, nHAC/PLGA/GO (0.5 wt%), nHAC/PLGA/GO (1.0 wt%) and nHAC/PLGA/GO (1.5 wt%)) were powdered to make a 100 mg/ml suspension. The experiment group with 100 ul suspension and control group with equal volume of DMEM complete medium were incubated for 24 h and were further incubated for 4 h after the CCK-8 was added to the incubator. The cell viability was obtained by measuring the absorbance at the wavelength of 450 nm using an ELISA reader. The cell viability was calculated by using the following formula,

$$ \mathrm{Cell}\  \mathrm{viability}\ \left(\%\right)=\left[\mathrm{A}\ \left(\mathrm{experiment}\right)-\mathrm{A}\ \left(\mathrm{blank}\right)\right]/\left[\mathrm{A}\ \left(\mathrm{control}\right)-\mathrm{A}\ \left(\mathrm{blank}\right)\right]\times 100\% $$

Where A (experiment) represents absorbance of wells with cells, CCK-8 solution and power samples solution; A (blank) represents absorbance of wells with medium and CCK-8 solution without cells and A (control) represents absorbance of wells with cells, CCK-8 solution without power samples solution.

### Statistical Analysis

Quantitative results were expressed as the mean value from at least triplicate samples ± standard deviation (SD). Student’s *t* test was used to the statistical analysis. A value of *p* < 0.05 was considered statistically significant. Data are marked ** to indicate *p* < <0.01.

## Additional file

Additional file 1: Figure S1. a Surface morphology of the nHAC. b EDS spectra of the nHAC. Figure S2 SEM images of a nHAC/PLGA; b nHAC/PLGA/GO (0.5 wt%). c nHAC/PLGA/GO (1.0 wt%); d nHAC/PLGA/GO (1.5 wt%) scaffolds. (DOCX 2689 kb)

## Abbreviations

3D: Three-dimensional
AFM: Atomic force microscopy
CCK-8: Cell counting kit-8
CTD: Computational topology design
DMEM: Dulbecco’s modified Eagle media
EDS: X-ray spectroscopy
FBS: Fetal bovine serum
FT-IR: Fourier transform infrared spectroscopy
GO: Graphene oxide
HA: Hydroxyapatite
MC3T3-E1: Osteoblast cells
MSCs: Mesenchymal stem cells
nHAC: Nano-hydroxyapatite/collagen
NIH-3T3: Fibroblast cells
PBS: Phosphate-buffered saline
PLA: Poly(lactic acid)
PLGA: poly(lactic-co-glycolic acid)
QNM-AFM: Quantitative nano-mechanical atomic force microscope
SD: Standard deviation
SEM: Scanning electron microscope
SFF: Olid free-form fabrication
XRD: X-ray diffraction
