# Supplementary material for: Graphene Oxide Hybridized nHAC/PLGA Scaffolds Facilitate the Proliferation of MC3T3-E1 Cells
Source: Nanoscale Res Lett. 2018 Jan 11;13:15. doi: 10.1186/s11671-018-2432-6 (PMC5764901; doi:10.1186/s11671-018-2432-6)
Supplement: Additional file 1: Figure S1. — a Surface morphology of the nHAC. b EDS spectra of the nHAC. Figure S2 SEM images of a nHAC/PLGA; b nHAC/PLGA/GO (0.5 wt%). c nHAC/PLGA/GO (1.0 wt%); d nHAC/PLGA/GO (1.5 wt%) scaffolds. (DOCX 2689 kb) [file 11671_2018_2432_MOESM1_ESM.docx]

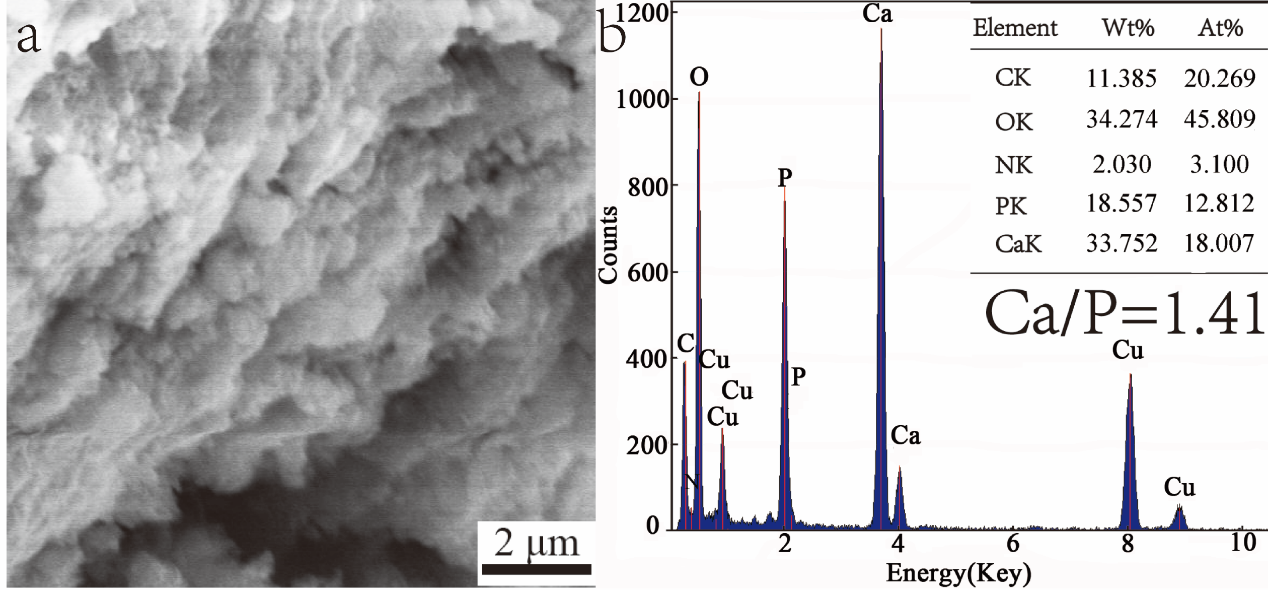


Figure S1 a) Surface morphology of the nHAC; b) EDS spectra of the nHAC;


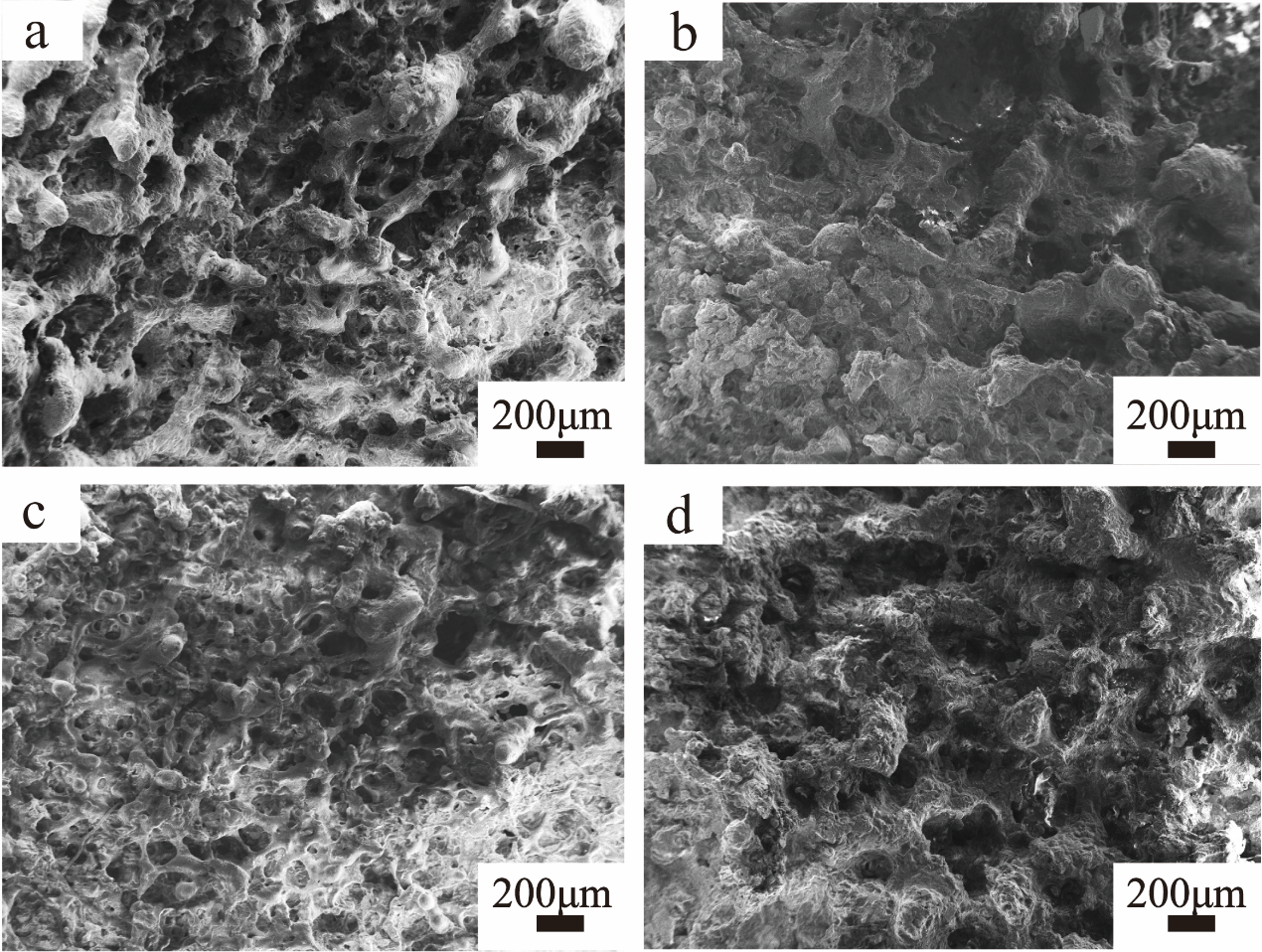


Figure S2 SEM images of a) nHAC/PLGA; b nHAC/PLGA/GO (0.5 wt%); c) nHAC/PLGA/GO (1.0 wt%); d) nHAC/PLGA/GO (1.5 wt%) scaffolds.
